# High Level of CD8^+^PD-1^+^ Cells in Patients with Chronic Myeloid Leukemia Who Experienced Loss of MMR after Imatinib Discontinuation

**DOI:** 10.3390/cells13080723

**Published:** 2024-04-22

**Authors:** Paulina Kwaśnik, Joanna Zaleska, Dorota Link-Lenczowska, Magdalena Zawada, Hubert Wysogląd, Bogdan Ochrem, Grażyna Bober, Ewa Wasilewska, Iwona Hus, Monika Szarejko, Witold Prejzner, Olga Grzybowska-Izydorczyk, Agnieszka Klonowska-Szymczyk, Ewa Mędraś, Michał Kiełbus, Tomasz Sacha, Krzysztof Giannopoulos

**Affiliations:** 1 Department of Experimental Hematooncology, Medical University of Lublin, 20-093 Lublin, Poland; kwasnik.paulina5@gmail.com (P.K.);; 2 Department of Hematology Diagnostics, Jagiellonian University Hospital in Kraków, 30-688 Kraków, Poland; 3 Department of Hematology, Jagiellonian University Hospital in Kraków, 30-688 Kraków, Poland; 4 Department of Hematooncology and Bone Marrow Transplantation, School of Medicine in Katowice, Medical University of Silesia, 40-032 Katowice, Poland; 5 Department of Hematology, Medical University of Białystok, 15-276 Białystok, Poland; 6 Department of Hematology, Institute of Hematology and Transfusion Medicine, 02-776 Warsaw, Poland; 7 Department of Clinical Transplantology, Medical University of Lublin, 20-093 Lublin, Poland; 8 Department of Hematology and Transplantology, Medical University of Gdańsk, 80-214 Gdańsk, Poland; 9 Department of Hematology, Medical University of Łódź, 93-513 Łódź, Poland; 10 Department of Hematology, Neoplastic Blood Disorders and Bone Marrow Transplantation in Wrocław, 50-367 Wrocław, Poland; 11 Chair of Hematology, Jagiellonian University Medical College in Kraków, 31-501 Kraków, Poland

**Keywords:** chronic myeloid leukemia (CML), treatment-free remission (TFR), discontinuation of imatinib, immune biomarker

## Abstract

Treatment-free remission (TFR) is achieved in approximately half of chronic myeloid leukemia (CML) patients treated with tyrosine kinase inhibitors. The mechanisms responsible for TFR maintenance remain elusive. This study aimed to identify immune markers responsible for the control of residual CML cells early in the TFR (at 3 months), which may be the key to achieving long-term TFR and relapse-free survival (RFS) after discontinuation of imatinib. Our study included 63 CML patients after imatinib discontinuation, in whom comprehensive analysis of changes in the immune system was performed by flow cytometry, and changes in the *BCR::ABL1* transcript levels were assessed by RQ-PCR and ddPCR. We demonstrated a significant increase in the percentage of CD8^+^PD-1^+^ cells in patients losing TFR. The level of CD8^+^PD-1^+^ cells is inversely related to the duration of treatment and incidence of deep molecular response (DMR) before discontinuation. Analysis of the ROC curve showed that the percentage of CD8^+^PD-1^+^ cells may be a significant factor in early molecular recurrence. Interestingly, at 3 months of TFR, patients with the e13a2 transcript had a significantly higher proportion of the PD-1-expressing immune cells compared to patients with the e14a2. Our results suggest the important involvement of CD8^+^PD-1^+^ cells in the success of TFR and may help in identifying a group of patients who could successfully discontinue imatinib.

## 1. Introduction

Tyrosine kinase inhibitors (TKI) transformed the treatment landscape of chronic myeloid leukemia (CML) for many years, extending patient life expectancy to levels comparable to age-matched healthy individuals [1]. Despite clear clinical benefits related to the use of TKI, a prolonged treatment time contributes to the incidence of adverse effects in many patients that can significantly reduce the quality of life. Furthermore, improved survival rates generate a high cost of the therapy, even when new generic substitutes are available [2,3]. The achievement of treatment-free remission (TFR) became an important objective of CML therapy [4]. Hitherto, clinical trials in the discontinuation of imatinib or second-generation TKI in first-line therapy in CML showed that more than 40% of patients remain in deep molecular response (DMR) and nearly all DMR and major molecular remission (MMR) loss occurs during the first 6 months after withdrawal. Upon re-initiation of therapy, over 90% of patients regain the initial depth of the molecular response [5]. However, the mechanisms that are responsible for TFR loss remain largely unexplained. There is a suggestion that maintaining long-term TFR is not directly related to the total disposal of the gene transcript *BCR::ABL1*, but that it could possibly result from immune surveillance restoration in CML [6,7].

The aim of the research was a complex analysis of changes in the immune system as well as *BCR::ABL1* gene transcript levels after imatinib discontinuation and at loss of MMR in CML patients, who previously achieved stable DMR.

## 2. Materials and Methods

### 2.1. Patients

Our study analyzed 63 patients with CML in chronic phase: 38 females (60.3%) and 25 males (39.7%) with a median age of 64 years (age range 25–87 years). To estimate the risk of survival, the newly developed EUTOS Long-Term Survival (ELTS) score, recommended by the ELN panel, was used [4]. According to the recommendations, ELTS score determines risk groups in patients much better than the existing Sokal, Euro, and EUTOS prognostic systems, especially among older patients.

### 2.2. Ethics Statement

The study was approved by the local Ethics Committee of the Medical University of Lublin (number KE-0254/174/2017) and all patients agreed to participate in the study of the Polish Adult Leukemia Study Group. Written consent from all patients was obtained.

### 2.3. Cell Isolation

Peripheral blood mononuclear cells (PBMC) were isolated by Biocoll (Biochrom, Berlin, Germany) density gradient centrifugation and cryopreserved at minus 80 °C until the time of analysis. The viability of obtained cells was planned at >95%, as determined by trypan blue staining (Sigma Aldrich, Saint Louis, MO, USA). The viable cells were quantified in a Neubauer chamber (Zeiss, Oberkochen, Germany). Samples were analyzed at the moment of imatinib discontinuation (month 0) and at 3 months after withdrawal.

### 2.4. Flow Cytometry for Detection of T Cells, B Cells, and Regulatory T Cells

One million PBMC were incubated with fluorochrome-labeled monoclonal antibodies (mAbs): anti-CD3-BV510, anti-CD4-PerCP, anti-CD8-FITC, anti-CD19-APC-Cy7, anti-CD25-PE-Cy7, anti-CD127-BV421 for 20 min at room temperature in the dark. All mAbs were purchased from Becton Dickinson (San Jose, CA, USA). To assess regulatory T cells, Human FOXP3 Fix/Perm Buffer Set (Becton Dickinson, San Jose, CA, USA) was used. After washing with stain buffer (Becton Dickinson, San Jose, CA, USA), cells were suspended in 2 mL of diluted buffer A and incubated for 10 min at room temperature in the dark. After 5 min of washing with stain buffer, cells were incubated for 30 min in 500 μL of prepared buffer C at room temperature in the dark. Next, the cells were washed with stain buffer and incubated with anti-FOXP3-PE mAb for 30 min at room temperature in the darkness. Approximately 100,000 stained cells of each sample were analyzed by flow cytometry using a FACSLyric (Becton Dickinson, San Jose, CA, USA). Data analyses were accomplished by FACS Suite software (version 1.5, Becton Dickinson, San Jose, CA, USA).

### 2.5. Flow Cytometry for Detection of Natural Killer (NK) Cells and Natural Killer T-Like (NKT-like) Cells

One million cells were incubated with fluorochrome-labeled mAbs: anti-CD45-BV510, anti-CD14-APC-H7, anti-CD3-PerCP, anti-CD56-PE-Cy7, anti-CD16-BV421, anti-iNKT-FITC, anti-CD161-PE for 20 min at room temperature in the dark. Next, the cells were washed twice with phosphate-buffered saline (PBS). A minimum of 100,000 cells were collected and analyzed.

### 2.6. Flow Cytometry for Detection of Myeloid Dendritic Cells (mDCs) and Plasmacytoid Dendritic Cells (pDCs)

One million cells were incubated with fluorochrome-labeled mAbs: lineage cocktail FITC (for exclusion: CD3, CD14, CD19, CD20, CD56), anti-HLA-DR-BV510, anti-CD123-PE-Cy7, anti-BDCA-1-PE, anti-BDCA-BV421 for 20 min at room temperature in the dark. The cells were washed twice with PBS. A minimum of 300,000 were measured and analyzed.

### 2.7. Assessment of PD-1 Expression Changes on Immune Cell Subpopulation by Flow Cytometry

Additionally, DC, NK, NKT-like, CD3^+^, CD4^+^, CD8^+^, and CD19^+^ cells were stained with fluorochrome-labeled mAb anti-PD-1-APC. The FMO (fluorescence minus one) control staining was performed to determine the boundary between cells positive and negative for the PD-1 molecule.

### 2.8. RNA Extraction

Total RNA was extracted according to Chomczyński and Sacchi protocol with double RNA chloroform extraction and 99.8% ethanol precipitation. The concentration and purity of isolated RNA were determined using the NanoDrop Lite spectrophotometer (Thermo Fisher Scientific, Waltham, MA, USA).

### 2.9. Reverse Transcription

To conduct quantitative analysis of *BCR::ABL1* gene expression by real-time quantitative reverse transcriptase polymerase chain reaction (RQ-PCR), a reverse transcription reaction was performed using SuperScript II Reverse Transcriptase (Thermo Fisher Scientific, Waltham, MA, USA) and Thermal Cycler BIOMETRA T-Personal 48 (Biometra, Göttingen, Lower Saxony, Germany).

In the case of droplet digital polymerase chain reaction (ddPCR), the reaction was carried out based on SuperScript™ VILO™ Reverse Transcriptase (Thermo Fisher Scientific, Waltham, MA, USA), according to BIO-RAD’s protocol, using the T100 Thermal Cycler (Bio-Rad, Hercules, CA, USA).

### 2.10. RQ-PCR for BCR::ABL1

RQ-PCR tests performed in the 7 parent institutions and laboratories certified according to the EUTOS (European Treatment and Outcome Study) regulations from Poland. The RQ-PCR method was performed according to European Leukemia Net (ELN) and EUTOS for CML standards [8].

### 2.11. ddPCR for BCR::ABL1

The ddPCR was performed at the Department of Hematology Diagnostics of Jagiellonian University Hospital in Kraków, Poland. The exact concentration of cDNA per reaction was determined experimentally. The cDNA was then amplified by ddPCR using two molecular probes: a probe labeled with a fluorescent dye FAM, specific for the *BCR::ABL1* fusion gene (for e13a2 and e14a2 variants), and a probe labeled with HEX dye specific for the *GUSB* reference gene [9]. In the first step of the ddPCR study, 20 µL of the reaction mixture was applied to the DG8 cartridge for drop generation. In the droplet generator, the cDNA was fractionated into 20,000 individual nanodroplets. The PCR reaction was then carried out in a thermocycler. The final stage was the analysis of nanodroplets in a drop reader, consisting of estimating the number of generated droplets and measuring the fluorescence of single cDNA molecules (a signal typical for the probe complementary to the *BCR::ABL1* gene fragment and for the probe complementary to the *GUSB* control gene). Then, on this basis, using the Poisson distribution, the absolute number of *BCR::ABL1* and *GUSB* gene copies needed to determine the level of expression of e13a2 and e14a2 transcripts was estimated.

### 2.12. Statistical Analysis

A Mann–Whitney U-test, a Wilcoxon matched-pairs signed-rank test, and a Kruskal–Wallis test were used to assess the differences between independent and dependent subgroups of patients. The correlations of variables were computed with the Spearman rank correlation coefficient. Non-normal distribution was found using the Shapiro–Wilk test. We used a significance level of *p* < 0.05 throughout the analysis. The confidence interval (CI) was 95%. The *p* values were adjusted using the Benjamini–Hochberg method [10]. Cell populations, for which the cohort-level difference in altered frequency, along with a statistical false discovery rate (FDR), were less than 0.05, were considered significant. The ROC (receiver operating characteristic) curve was used to examine the model to determine the strongest immune predictor. The area under the curve (AUC) provides a measure of overall predictive accuracy. The logistic regression was used to estimate odds ratio (OR) and 95% CI as approximations of relative risks, with adjustment for potentially important confounding variables deemed significant in a preliminary exploration of the data. All statistical analyses were performed using Statistica 13.0 Software and GraphPad Prism 8 Software.

## 3. Results

### 3.1. Patient Characteristics

The clinical characteristics of the patients are summarized in detail in Table 1.

### 3.2. Characteristics of the Immune System of CML Patients 3 Months after Imatinib Discontinuation

**Figure 1:**
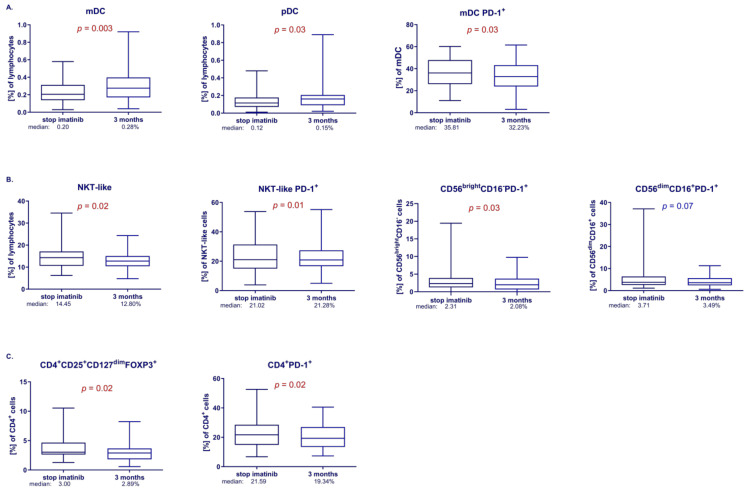
Immunological characteristics of all patients enrolled in the study. Differences in the percentages of immune populations at the moment of stopping imatinib and at 3 months after discontinuation in analyzed patients (Wilcoxon signed-rank test). (**A**) DC subpopulations; mDC, median: 0.20 vs. 0.28%; pDC, median: 0.12 vs. 0.15%; mDC PD-1^+^, median: 35.81 vs. 32.23%. (**B**) NKT-like and NK cells; NKT-like cells, median: 14.45 vs. 12.80%; NKT-like PD-1^+^; median: 21.02 vs. 21.28%; CD56^bright^CD16-PD-1^+^, median: 2.31 vs. 2.08%; CD56^dim^CD16^+^PD-1^+^, median: 3.71 vs. 3.49%. (**C**) CD4^+^ T cells and regulatory T cells; Treg CD4^+^CD25^+^CD127^dim^FOXP3^+^, median: 3.00 vs. 2.89%; CD4^+^PD-1^+^, median: 21.59 vs. 19.34%. Red color indicates statistical significance *p* < 0.05, blue color indicates tendency *p* < 0.1.

Multicolor flow cytometry was managed to analyze the populations of immune cells that could be responsible for the restoration of immune system control over the CML clone after discontinuation of treatment. General immunological characteristics of patients were obtained by analyzing all patients after imatinib discontinuation (Figure 1). Analyses in pairs showed a significant increase in the percentage of mDCs (median 0.20 vs. 0.28%, *p* < 0.05; FDR < 0.05) and pDCs (median 0.12 vs. 0.15%, *p* < 0.05; FDR < 0.05), and a significant decrease in mDC PD-1^+^ (median 35.81 vs. 32.23%, *p* < 0.05; FDR < 0.05) at 3 months after treatment discontinuation for all patients (Figure 1A). There were no significant differences in PD-1 expression on pDCs at 0 vs. at 3 months.

There was a significantly reduced level of NKT-like cells (median 14.45 vs. 12.80%, *p* < 0.05; FDR < 0.05), a significant increase in NKT-like PD-1^+^ cells (median 21.02 vs. 21.28%, *p* < 0.05; FDR < 0.05) and a significant downregulation of CD56^bright^CD16^−^PD-1^+^ cells (median 2.31 vs. 2.08%, *p* < 0.05; FDR < 0.05) at 3 months after discontinuation. We observed a downward trend in the percentage of CD56^dim^CD16^+^PD-1^+^ cells (median 3.71 vs. 3.49%, *p* < 0.07; FDR < 0.07) at 3 months in comparison with baseline samples (Figure 1B). There were no significant differences in frequencies of NK cells between month 0 and at 3 months.

Analysis of regulatory lymphocytes T showed a significant decrease in frequencies of CD4^+^CD25^+^CD127^dim^FOXP3^+^ (median 3.00 vs. 2.89%, *p* < 0.05; FDR < 0.05) and a significant decrease in the percentage of CD4^+^PD-1^+^ cells (median 21.59 vs. 19.34%, *p* < 0.05; FDR < 0.05) at 3 months compared to month 0 (Figure 1C). There were no significant differences in frequencies of CD8^+^ and CD19^+^ cells in patients after withdrawal between month 0 and at 3 months.

Characteristics of the immune system of patients after imatinib discontinuation at month 3 of TFR included the following:

- A significant increase in the percentages of dendritic cell subpopulations (mDC and pDC),
- A significant decrease in the percentages of cells positive for the PD-1 molecule, including DC subpopulations (mDC PD-1^+^), NK cells (CD56^dim^CD16^+^PD-1^+^, CD56^bright^CD16^−^PD-1^+^), and CD4^+^ helper lymphocytes (CD4^+^PD-1^+^),
- A significant decrease in the percentages of regulatory lymphocytes (CD4^+^CD25^+^CD127^dim^FOXP3^+^), and NKT-like lymphocytes.

### 3.3. Immunological Differences between CML Patients in Remission and Patients with Molecular Recurrence

There were changes present in the immune system in relation to *BCR::ABL1* transcript levels measured by the ddPCR. Comparing the results of the *BCR::ABL1* transcript obtained by ddPCR and RQ-PCR, we observed a strong correlation between the results of these methods. Studies indicate its high sensitivity and accuracy as well as a good correlation with RQ-PCR results [11].

**Figure 2:**
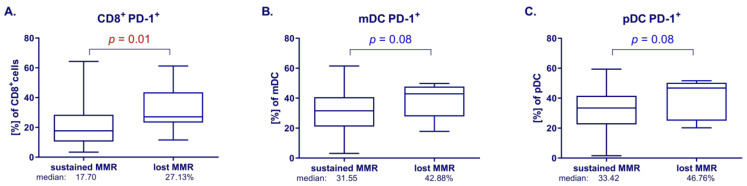
Changes in immune populations in patients with stable MMR or lost MMR at 3 months after withdrawal. Differences in the percentages of immune populations between patient groups were analyzed with the Mann–Whitney U test. (**A**) CD8^+^PD-1^+^ cells, median: 17.70 vs. 27.13%. (**B**) mDC PD-1^+^, median: 31.55 vs. 42.88%. (**C**) pDC PD-1^+^, median: 33.42 vs. 46.76%. Red color indicates statistical significance *p* < 0.05, blue color indicates tendency *p* < 0.1.

Based on *BCR::ABL1* transcript quantification analysis, the cohort of patients was divided into patients maintaining or losing MMR, and patients maintaining or losing DMR. The changes in the immune system described above were analyzed to determine whether they are related to the loss or maintenance of MMR (Figure 2).

The analysis showed statistically significant changes in the percentage of CD8^+^PD-1^+^ depending on MR status. In patients who experienced lost MMR (molecular recurrence) at 3 months after imatinib discontinuation, a significant increase in CD8^+^PD-1^+^ cells was observed compared to patients who maintained MMR (median 27.13 vs. 17.70%, *p* < 0.05; FDR < 0.05) (Figure 2A). These significant changes are already noticeable in patients who have lost the molecular response at the DMR level (median 27.78 vs. 17.83%, *p* < 0.05; FDR < 0.05).

After 3 months of TFR, we also noticed an upward trend in the percentage of mDC PD-1^+^ cells in the patients with molecular recurrence compared to the patients with sustained MMR (median 42.88 vs. 31.55%, *p* < 0.10; FDR < 0.10) (Figure 2B). Analogous observations were noted regarding pDC PD-1^+^: there was a tendency to increase the percentage of pDC PD-1^+^ in patients with lost MMR after 3 months compared to patients with stable MMR (median 46.76 vs. 33.42%, *p* < 0.10; FDR < 0.10) (Figure 2C).

**Figure 3:**
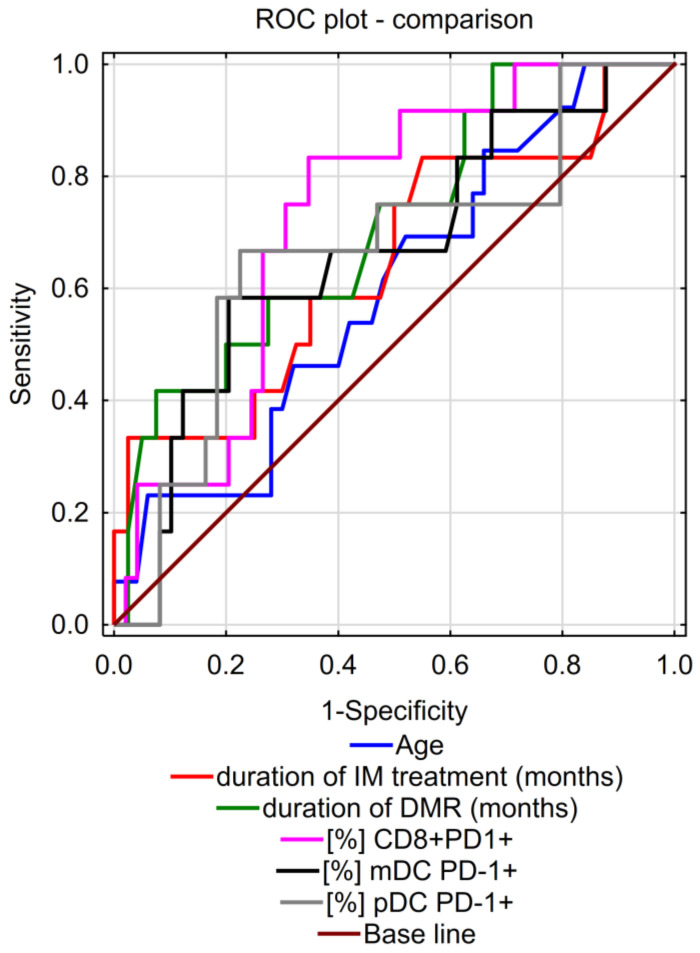
Comparison of ROC curves showing an analysis of potential immunological biomarkers by age, duration of DMR, length of TKI treatment before TFR, and percentages of CD8^+^PD-1^+^, mDC PD-1^+^, and pDC PD-1^+^. Among the clinical data evaluated, ROC analysis indicates CD8^+^PD-1^+^ percentage as the strongest factor in early molecular reoccurrence (lost MMR) (*p* < 0.01). The statistically significant marker of lost MMR is the duration of DMR before stopping treatment (*p* < 0.05) (ROC curves).

Analysis of the ROC curve including age, duration of DMR response, length of TKI treatment before discontinuation, and percentages of CD8^+^PD-1^+^, mDC PD-1^+^, and pDC PD-1^+^, showed that the percentage of CD8^+^PD-1^+^ cells is the strongest predictor of early lost MMR among other analyzed parameters (Figure 3). A higher likelihood of molecular recurrence is associated with a higher percentage of CD8^+^PD-1^+^ cells after 3 months of imatinib withdrawal (AUC = 0.73, 95% CI: 0.59 to 0.87, *p* = 0.001). The duration of DMR before discontinuation may also be an important predictor. A higher likelihood of lost MMR is correlated with the shorter duration of DMR (AUC = 0.71, 95% CI: 0.55 to 0.87, *p* = 0.012). The presented data suggest that the younger the age and the shorter the treatment period before TFR, the more frequently molecular recurrence was observed, but no significant differences were observed (age: AUC = 0.60, 95% CI: 0.43 to 0.76, *p* = 0.2549; duration of treatment: AUC = 0.65, 95% CI: 0.46 to 0.84, *p* = 0.123).

**Figure 4:**
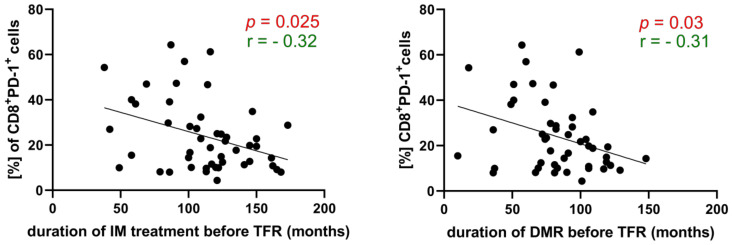
Correlation between percentage of CD8^+^PD-1^+^ in patients at 3 months after withdrawal, and duration of treatment and DMR before TFR trial. The percentage of CD8^+^PD-1^+^ is inversely related to the treatment of imatinib (R = −0.3227, 95% CI: −0.5618 to −0.03378) and DMR duration (R = −0.3118, 95% CI: −0.5535 to −0.02176) (Spearman r). Red color indicates statistical significance *p* < 0.05, green color indicates the correlation coefficient.

Spearman r correlation showed negative correlations between the percentage of CD8^+^PD-1^+^ at 3 months after discontinuation, and both the length of treatment (R = −0.32, 95% CI: −0.56 to −0.034, *p* < 0.05) and the duration of DMR (R = −0.31, 95% CI: −0.55 to −0.02, *p* < 0.05) (Figure 4).

Additionally, we found positive relationships between the duration of imatinib therapy and duration of DMR (R = 0.65, 95% CI: 0.41 to 0.80, *p* < 0.0001).

Logistic regression analysis was performed, which showed that the percentage of CD8^+^PD-1^+^ cells had a statistically significant impact on the risk of early lost MMR, which was confirmed by the Wald chi-square statistic and the odds ratio (OR). The analysis included 61 important cases, of which molecular recurrence occurred in 12 cases (19.67%) and did not occur in 49 cases (80.33%). The chi-square statistic is statistically significant, which means that introducing the variable percentage of CD8^+^PD-1^+^ cells into the model provides new information on the factors in MMR loss during the TFR trial. The OR indicates that an increased CD8^+^PD-1^+^ percentage increases the risk of early lost MMR by an average of 15.66 times (OR = 15.66, 95% CI: 1.13 to 216.22).

Based on the logistic regression analysis performed, we can build a model in the form: logit p = −2.58 + 0.05 * percentage of CD8^+^PD-1^+^.

Related to the developed model, 8.33% of cases with molecular recurrence and 97.96% without molecular recurrence were correctly classified, i.e., 80.33% of patients were correctly classified based on the developed model (Supplementary Figure S1). There is over 4 times’ greater chance (OR = 4.36) for the correct classification of the patient based on the developed model.

Other correlations we observed between the analyzed immune cell populations and two-time pointsare summarized in Supplementary Figure S2.

Analyses comparing patients with MMR loss and those with stable MMR after 3 months of TFR suggest that cell populations expressing PD-1 may be important in maintaining TFR. The percentage of CD8^+^PD-1^+^ may be a biomarker of early molecular recurrence and is inversely related to the duration of treatment and incidence of DMR before discontinuation. According to our results, the loss of MMR is related to the length of DMR before discontinuation. As reported in other studies, the length of DMR is correlated with the duration of imatinib treatment.

### 3.4. Other Clinical Parameters in CML Patients in TFR

Other clinical parameters were assessed, including *BCR::ABL1* transcript type, withdrawal syndrome (WS), and ELTS score, which are suggested to be important in the TFR trial. We examined whether these parameters influence the modulation of the immune system, which is crucial in the success of the withdrawal trial.

#### 3.4.1. Type of Transcripts e13a2 and e14a2

**Figure 5:**
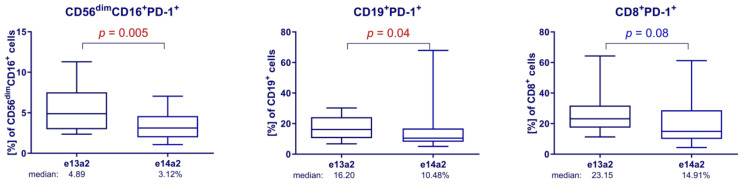
Analysis of changes in the immune system of patients depending on the transcript type of *BCR::ABL1*. Higher percentage of the population expressing PD-1 in patients with e13a2 compared to patients with e14a2 (Mann–Whitney U test). Differences between patients with e13a2 vs. patients with e14a2: CD56^dim^CD16^+^PD-1^+^, median: 4.89 vs. 3.12%; CD19^+^PD-1^+^, median: 16.20 vs. 10.48%; CD8^+^PD-1^+^, median: 23.15 vs. 14.91%. Red color indicates statistical significance *p* < 0.05, blue color indicates tendency *p* < 0.1.

The analysis of alterations in the immune system about the *BCR::ABL1* transcript type showed that at 3 months of TFR, the patients with e13a2 transcript had significantly higher values of CD56^dim^CD16^+^PD-1^+^ cells (median 4.89 vs. 3.12%, *p* < 0.01; FDR < 0.02) and CD19^+^PD-1^+^ cells (median 16.20 vs. 10.48%, *p* < 0.05; FDR < 0.07), and a tendency for higher values of the percentage of CD8^+^PD-1^+^ cells (median 23.15 vs. 14.91%, *p* < 0.09; FDR < 0.09), when compared to patients with e14a2 transcripts (Figure 5). Interestingly, in ddPCR we found a significantly higher transcript level in patients with e13a2 transcript type as compared to patients with e14a2 at 3 months after imatinib discontinuation.

The poorer results of patients with the e13a2 transcript type may be related to biological aspects of tyrosine kinase activity and thus have an immunomodulatory effect on immune cells. The e13a2 transcript was associated with an increase in the number of cells expressing PD-1, which may lead to impaired immune response. In patients with e13a2, there was an increased rise in the percentage of NK cells, B lymphocytes, and CD8+ lymphocytes.

#### 3.4.2. Withdrawal Syndrome (WS)

We observed changes in the immune system between patients who experienced WS and those without WS at 3 months after discontinuation of imatinib. We showed that patients who developed the symptoms had a significantly lower percentage of CD56^dim^CD16^+^PD-1^+^ NK cells (median 2.35 vs. 4.69%, *p* < 0.001; FDR < 0.0.001) and a downward trend in levels of NKT-like PD-1^+^ cells (median 19.46 vs. 21.96 %, *p* = 0.09; FDR = 0.09) compared to patients without WS (Figure S3). There were no significant differences between the occurrence of WS and *BCR::ABL1* transcript level.

The analysis of patients with WS showed a decrease in the percentage of NK cells positive for the PD-1 molecule (CD56^dim^CD16^+^PD-1^+^), which may indicate the unblocking of the cytotoxic function of NK cells and the release of cytokines, which are suggested to cause the symptoms of WS.

#### 3.4.3. ELTS Scores

Patients with intermediate/high ELTS scores had significantly reduced percentages of DC subpopulations (median of mDC 0.32 vs. 0.17%, *p* < 0.05; FDR < 0.05; median of pDC 0.17 vs. 0.08%, *p* < 0.05; FDR < 0.05), but a significantly increased pDC with expression PD-1^+^ relative to patients with a low ELTS score (median 31.44 vs. 44.53%, *p* < 0.05; FDR < 0.05). Patients with an intermediate/high ELTS score had a significantly lower proportion of NK cells compared to patients with a low ELTS score (median of CD56^bright^CD16^−^ cells: 0.40 vs. 0.22%, *p* < 0.05; FDR < 0.05), and a significantly higher percentage of the populations with PD-1 expression (median of NKT-like PD-1^+^ cells 18.75 vs. 33.93%, *p* < 0.01; FDR < 0.01, median of CD4^+^PD-1^+^ 18.30 vs. 28.26%, *p* < 0.05; FDR < 0.05) (Figure S4). There were no significant differences in *BCR::ABL1* levels between patients with different ELTS scores.

Patients with intermediate/high ELTS scores had lower percentages of dendritic cell subpopulations (mDC and pDC), higher percentages of cells expressing PD-1 (pDC PD-1^+^, NKT-like PD-1^+^, CD4^+^PD-1^+^) and lower percentages of NK cells (CD56^bright^CD16^−^).

## 4. Discussion

The clinical course of CML is characterized by a deep impairment of the immune response against leukemia cells. It has been suggested that the successful TFR is due to regaining immune surveillance of residual leukemia cells [12,13,14]. The objective of this research was to determine the crucial immune indicators that play a role in eradicating or managing remaining CML cells during the early stages of TFR (at 3 months). Accordingly, studies have shown that in the first months of treatment with imatinib, the number of DCs returns to near-normal levels [15]. Moreover, the amount and functionality of pDCs are restored in patients achieving a DMR during imatinib treatment [16]. The increase in DC percentage in patients in TFR observed in our study may represent the characteristic of the recovery of the immune system of CML patients to a comparable to healthy population with the ability to control leukemic cells. Mohty et al. [16] reported that patients in DMR after imatinib treatment achieved pDC levels comparable to healthy people, which is aligned with our observation. Some studies have shown that a limited number of pDCs can induce anti-leukemic immunity early in the evolution of CML [17].

Our study revealed substantial changes in PD-1 expression in the immune system cells of CML patients during imatinib therapy and after treatment discontinuation. The constitutive expression of PD-1 on effector cells induces a coinhibitory signal, resulting in aberrant PD-1 signaling that promotes apoptosis, anergy, and functional exhaustion of CD8^+^ cells [18]. Brück et al. [19] described increased PD-1 expression in the leukemic bone marrow microenvironment. They showed that a high percentage of PD1^+^TIM3^−^CD8^+^ T cells is associated with a lower likelihood of DMR. Mumprecht et al. [20] also reported a high percentage of CD8^+^PD-1^+^ lymphocyte fraction in CML patients in the chronic phase. Changes in PD-1 expression on T cells were described by Hughes et al. [21], who observed a decrease in percentages of CD4^+^PD-1^+^ and CD8^+^PD-1^+^ in patients with MR4.5, which further correlated with the increased immunological function of T and NK cells. A decrease in PD-1 expression may restore the proper functioning of CD8^+^ lymphocytes and thus promote a successful TFR. PD-1/PD-L1 interactions might contribute to the inhibition of NK and T cells failing the minimal residual disease elimination, and may be associated with relapse of leukemia. PD-1/PD-L1 blockade could restore specific functions of the CD8^+^ CTL or NK that might be important in controlling CML progression [20,22]. The significance of PD-1 expression on NK cells is particularly important in immunity against low or no MHC-expressed tumors which NK cells recognize in contrast to T cells [23]. Recent studies showed patients with low or no MHC expression tumors who respond to anti-PD-1 therapy, emphasizing that cells other than cytotoxic T cells also express PD-1, and could be responsible for anti-tumor immunity [24,25]. PD-L1 and PD-L2 are expressed on DC cells but also T cells and tumor cells [26,27]. Thus DCs are capable of bidirectional T cell-inhibitory signaling by the expression of ligands for PD-1, as well as the PD-1 itself [28]. Moreover, it has been shown that DC PD-1^+^ has survival difficulty, constitutes mainly immature populations, and produces fewer pro-inflammatory cytokines [29,30].

Research has shown that after imatinib [31,32,33] and the second generation of TKI [34,35], the e14a2 transcript is associated with a faster and deeper molecular response, which results in better TFR rates [36,37,38]. The e14a2 transcript is also associated with an improved probability of event-free and blast-free survival [34]. Recent studies focus on analyzing these differences, which are associated with beneficial responses to treatment in patients with the e14a2 transcript. Newly published data indicate that the RT-qPCR method has certain limitations in estimating distinct e13a2 and e14a2 transcripts [9,39,40]. Due to the different sizes of the verified amplicons and slight differences in PCR amplification efficiency, the e14a2 transcript has a lower amplification efficiency, which may explain the overestimation of e13a2 and, therefore, part of the observed differences in treatment response. However, these differences, as described [39,41], are small considering the variability in test results and do not affect overall survival, but it can be expected that they may have some importance in qualifying patients for a withdrawal trial. Perhaps the ddPCR technique, which is insensitive to differences in amplification kinetics, will be more widely used in the clinic and will help eliminate this problem [42,43]. However, the molecular response of patients may be due to biological differences in the tyrosine kinase encoded by the e13a2 and e14a2 transcript [44].The e13a2 transcript results in increased tyrosine kinase activity. The enhanced responses to TKI in patients with the 14a2 transcript may be associated with decreased tyrosine kinase activity due to an additional 25 amino acids in the e14a2 transcripts, which determine changes in the structure of the BCR::ABL1 kinase binding domain [45]. As a result, some studies show a reduced number of platelets [34,45] and an increased white blood cell count in patients with the e13a2 transcript [46]. It may suggest that the type of *BCR::ABL1* transcript could impact the immune response. Perhaps, better TFR rates in patients with e14a2 transcript may be related to immune changes, e.g., lower percentages of NK and CD19^+^ cells with PD-1 expression, which does not block the function of these cells and thus allows the immune control of residual leukemic cells. This is in line with previous studies that showed that the e14a2 transcript was more immunogenic and elicited a stronger effect against host CML [47,48]. It is necessary to investigate the biological mechanisms responsible for the differences in tyrosine kinase activity determined by different types of transcripts.

The importance of immune cell involvement during TFR also suggests the occurrence of specific symptoms in some patients after discontinuation of TKI treatment. Some of these patients experience diffuse mild to moderate musculoskeletal pain, known as “withdrawal syndrome” (WS). WS affects approximately 40% of patients after discontinuation of TKI therapy [5,49,50]. A comparable percentage of patients with WS as in our research was recently observed in a study by Petrov et al. [50]. Some patients may even require re-treatment of TKI due to pain caused by WS symptoms [51]. In our study, one patient restarted treatment due to WS. The phenomenon of WS is probably caused by the release of cytokines resulting from the unblocking of tyrosine kinase after discontinuation of its inhibitor. This approach is justified by the observation that the occurrence of WS is associated with a higher probability of sustaining molecular remission [52]. There is evidence to suggest this is probably correlated with the longer duration of TKI treatment [49]. Unblocking of immunocompetent cells, which may be important in immune surveillance of the leukemic cell clone, may also cause the symptoms mentioned. Importantly, in our study we observed that NK cells may play a role in the occurrence of WS, among which the fraction of cells with the PD-1 molecule decreases after discontinuation of imatinib. The decrease in PD-1 expression on NK cells suggests a return to their normal functional and morphological state, which restores the cytotoxic function of CD56^dim^CD16^+^ cells. It can be speculated that the sudden unblocking of NK cell function causes the release of proinflammatory cytokines and chemokines, resulting in inflammation and, consequently, the occurrence of WS. Moreover, NK cell activation, along with the biological effects of c-Kit tyrosine kinase receptor inhibition through changes in pain sensitivity, may also cause symptoms of WS [53], which in turn may drive inflammation through the effects on mast cells. Restoration of the function of CD56^dim^CD16^+^ NK cells may also be of importance in the proven lower risk of MMR loss in patients with WS [52].

The success of TFR is associated with a longer duration of TKI treatment and a DMR, which has been proven in many studies [54,55,56,57,58]. In our study, we confirmed that a longer duration of DMR before stopping treatment resulted in a lower amount of *BCR::ABL1* and thus may subsequently prevent molecular recurrence of the disease. Furthermore, we have shown that the duration of imatinib treatment correlates with the duration of DMR. Our results indicate that both longer duration of treatment and longer duration of DMR are associated with a lower percentage of the CD8^+^ population expressing PD-1, whereas only DMR duration and percentage of CD8^+^PD-1^+^ cells can be effective predictors of MMR loss and contribute to early molecular recurrence.

The significant alterations in the percentages of the analyzed immune cell populations indicate the importance of the contribution of immunocompetent cells in maintaining the CML patients in TFR. The characterization of the immune system, which likely plays the principal role in achieving long-term RFS and TFR, may aid in identifying the group of patients who could safely discontinue imatinib. However, this necessitates further study of immune changes in TFR and also long-term observations.

## Figures and Tables

**Table 1:** Characteristics of CML patients in this study.

Characteristic	No. of Patients (%) or Median [Range]
*N*	63
Age (years)	64 [25–87]
Gender	
Female	38 (60.3)
Male	25 (39.7)
ELTS score	
Low	32 (78.1)
Intermediate	7 (17.1)
High	2 (4.8)
Sokal score	
Low	39 (76.5)
Intermediate	6 (11.8)
High	6 (11.8)
Transcript type ^a^	
e14a2	34 (65.4)
e13a2	15 (28.8)
e13a2 and e14a2	3 (5.8)
MR at the moment of withdrawal ^b^	
MR4.0	2 (3.3)
MR4.5 or more	58 (96.7)
The median duration of treatment (months)	115 [38–173]
The median duration of DMR before discontinuation (months)	81.5 [10–148]
Occurrence of withdrawal syndrome ^c^	21 (42.0)
No. of patients with DMR loss in 3 months ^b^	24 (38.1)
No. of patients with MMR loss in 3 months ^b^	13 (20.6)

Abbreviations: DMR, deep molecular response; ELTS score, EUTOS Long-Term Survival score; MMR, major molecular response. ^a^ based on RQ-PCR; ^b^ based on ddPCR; ^c^ caused treatment resumption in one patient.

## Data Availability

The data that support the findings of our study are available on request from the corresponding author.

## Supplementary Materials

The following supporting information can be downloaded at: https://www.mdpi.com/article/10.3390/cells13080723/s1, Figure S1: Graphical representation of correlation matrix; Figure S2: Immunological observation in patients with or without symptoms of withdrawal syndrome (WS); Figure S3: Immunological characteristics of patients classified according to the ELTS scores.
