# Supplementary material for: High Level of CD8+PD-1+ Cells in Patients with Chronic Myeloid Leukemia Who Experienced Loss of MMR after Imatinib Discontinuation
Source: Cells. 2024 Apr 22;13(8):723. doi: 10.3390/cells13080723 (PMC11048908; doi:10.3390/cells13080723)
Supplement: Supplementary file 1 [file cells-13-00723-s001.zip › cells-2964460-supplementary.pdf]

	Classification of Cases (Arkusz1 in CML discontinuation of imatinib) Odds ratio: 4,3636 Perc. correct: 80,33%		
Observed	Pred. yes	Pred. no	Percent Correct
yes	48	1	97,95918
no	11	1	8,33333

*Figure S1 Classification of cases & odds ratio. The odds ratio (OR) indicate the impact of CD8$^+$PD-1$^+$ percentage on the risk of early MMR loss after imatinib discontinuation. This model was developed: logit p = -2.58 + 0.05 * percentage of CD8$^+$PD-1$^+$. Based on the created model, there is over than 4 times greater chance (OR=4.36) for the correct classification of the patient.*

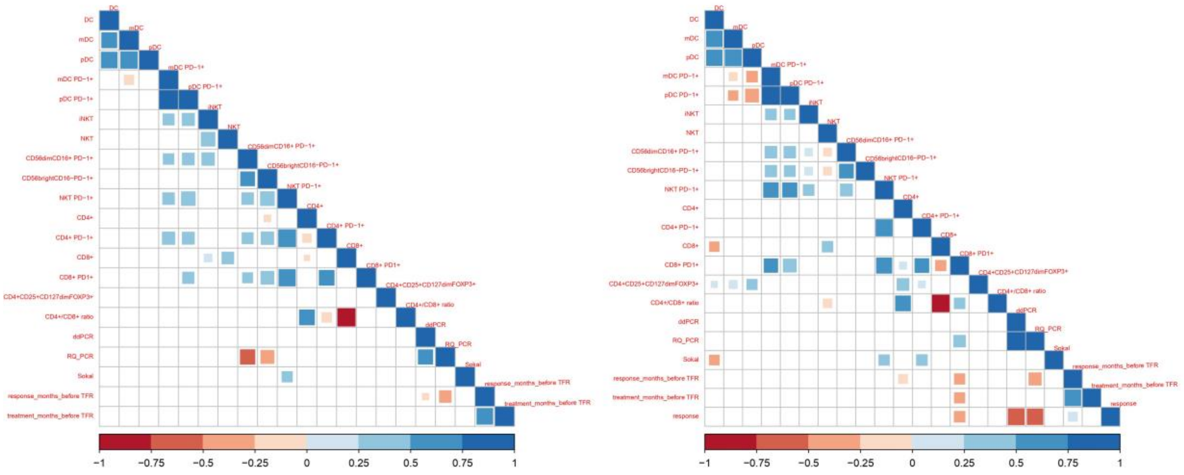

Figure S2. Graphical representation of correlation matrix. The correlogram shows statistical relationships between the quantity of investigated cells' populations and clinical features in CML patients revealed by the Spearman test. Positive correlations are displayed in blue and negative correlations in red color, moreover, color intensity and the square sizes are proportional to the correlation coefficient values. Only significant results were shown; the baseline was confirmed as P < .05. Moment of withdrawal (the moment of stopping imatinib) (left figure); at 3 months of TFR (right figure); the 'response' category corresponds to revealing MR3.0 at least (Spearman r).

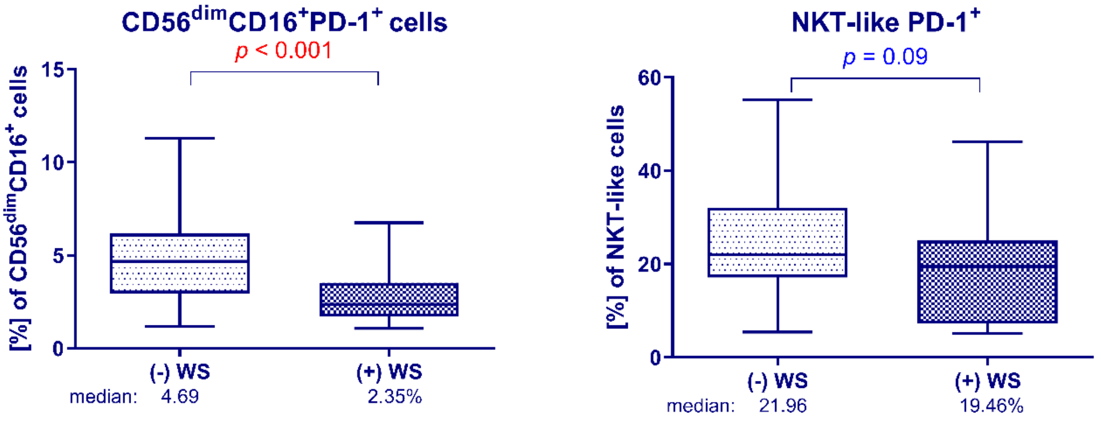

Figure S3. Immunological observation in patients with or without symptoms of withdrawal syndrome (WS). Differences in the percentages of immune populations between patients without (-)WS and patients with (+)WS at 3 months of TFR (Mann-Whitney U test): CD56^{dim}CD16$^+$PD-1$^+$, median: 4.69 vs 2.35%; NKT-like PD-1$^+$,

median: 21.96 vs 19.46%. Red color indicates statistical significance $p < 0.05$, blue color indicates tendency $p < 0.1$.

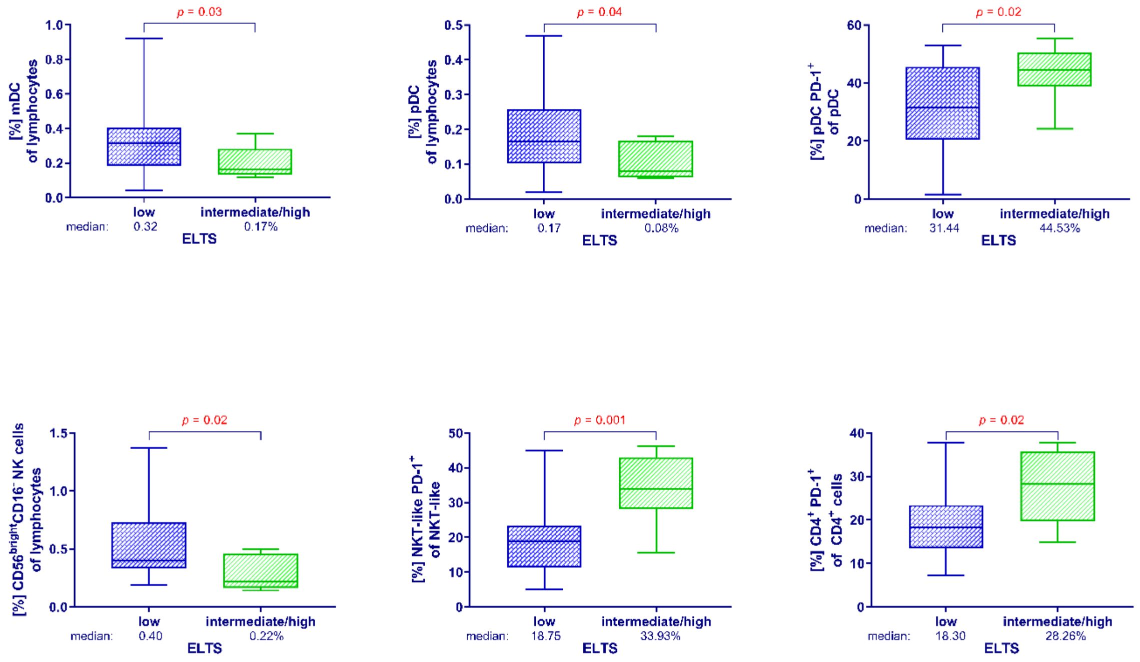

Figure S4. Immunological characteristics of patients classified according to the ELTS scores. Differences between the levels of DC, NK cells and populations with PD-1^{+} in patients with low ELTS compared to patients with intermediate/high ELTS scores at 3 months (Mann-Whitney U test): mDC, median: 0.32 vs 0.17%; pDC, median: 0.17 vs 0.08%; pDC PD-1^{+}, median: 31.44 vs 44.53%; CD56^{bright}CD16^{-}, median: 0.40 vs 0.22%; NKT-like PD-1^{+}, median: 18.75 vs 33.93%; CD4^{+}PD-1^{+}, median:18.30 vs 28.26%. Red color indicates statistical significance $p < 0.05$, blue color indicates tendency $p < 0.1$.
